# The Role of Green Building Materials in Reducing Environmental and Human Health Impacts

**DOI:** 10.3390/ijerph17072589

**Published:** 2020-04-10

**Authors:** Seyed Meysam Khoshnava, Raheleh Rostami, Rosli Mohamad Zin, Dalia Štreimikienė, Abbas Mardani, Mohammad Ismail

**Affiliations:** 1 UTM Construction Research Centre, Institute for Smart Infrastructure and Innovative Construction, School of Civil Engineering, Faculty of Engineering, Universiti Teknologi Malaysia (UTM), Johor 81310, Malaysia; kseyedmeysam@utm.my; 2 Department of Architecture, Sari Branch, Islamic Azad University, Sari 4816119318, Iran; raheleh.rostami@gmail.com; 3 School of Civil Engineering, Faculty of Engineering, Universiti Teknologi Malaysia (UTM), Johor 81310, Malaysia; roslizin@utm.my (R.M.Z.); mohammad@utm.my (M.I.); 4 Lithuanian Institute of Agrarian Economics, A. Vivulskio g. 4A-13, 03220 Vilnius, Lithuania; 5 Informetrics Research Group, Ton Duc Thang University, Ho Chi Minh City 758307, Vietnam; 6 Faculty of Business Administration, Ton Duc Thang University, Ho Chi Minh City 758307, Vietnam

**Keywords:** sustainable development, green building materials, biocomposite, life cycle assessment, human health, volatile organic compounds

## Abstract

Conventional building materials (CBMs) made from non-renewable resources are the main source of indoor air contaminants, whose impact can extend from indoors to outdoors. Given their sustainable development (SD) prospect, green building materials (GBMs) with non-toxic, natural, and organic compounds have the potential to reduce their overall impacts on environmental and human health. In this regard, biocomposites as GBMs are environmentally friendly, safe, and recyclable materials and their replacement of CBMs reduces environmental impacts and human health concerns. This study aims to develop a model of fully hybrid bio-based biocomposite as non-structural GBMs and compare it with fully petroleum-based composite in terms of volatile organic compound (VOC) emissions and human health impacts. Using a small chamber test (American Society for Testing and Materials (ASTM)-D5116) for VOC investigation and SimaPro software modeling with the ReCiPe method for evaluating human health impacts. Life cycle assessment (LCA) methodology is used, and the results indicate that switching the fully hybrid bio-based biocomposite with the fully petroleum-based composite could reduce more than 50% impacts on human health in terms of indoor and outdoor. Our results indicate that the usage of biocomposite as GBMs can be an environmentally friendly solution for reducing the total indoor and outdoor impacts on human health.

## 1. Introduction

The construction industry (CI) is one of the firmest emergent sectors in rapid urbanization due to the increasing population in urban areas [1]. This urban population has rapidly grown from 751 million (1950) to 4.2 billion (2018) in the world [2]. The affluence of this urbanization makes this industry the most astonishing consumer of materials, most of them from non-renewable resources that need replenishing [3]. The sustainable development (SD) perspective for construction materials is the effective manner of resources usage to meet the demands and preconditions of existing and future generations while reducing environmental degradation [4,5]. There is global concern and awareness about hazards from conventional building materials (CBMs) that have both social and environmental impact [6]. Quantifying these overall impacts on environmental and human health is complex and currently unaccounted. For instance, toxic materials that affect indoor air quality (IAQ), produce toxicity pollution for the environment and human during the production stage [7]. Reduction in different characters of building materials such as embodied energy [8], energy consumption [9], CO_2_ emission [10], and recyclability [11] can simultaneously affect both environmental and human health. To address these issues, new materials and technologies are urgently needed in the construction industry [12].

Above and beyond, CBMs are major contributors to indoor emission sources of volatile organic compounds (VOCs) that have the potential to deteriorate IAQ [13]. In terms of IAQ, most programs for evaluation materials such as the U.S. Green Building Council of Leadership in Energy and Environmental Design (LEED) rating system are concentrated on VOC prevention. In this regard, there are several standard tests, such as the small chamber test (American Society for Testing and Materials (ASTM)-D5116), for evaluating the emission of indoor VOCs. This apprehension of the health impacts of CBMs can extend from indoor to outdoor quality. Environmental burdens and human health impacts exist throughout the life of CBMs from non-renewable resources [14,15]. It is important to realize and simplify the complex reality effects of CBMs in the life cycle, especially the impact on human health, which is crucial. The life cycle assessment (LCA) is a common methodology for measuring the environmental weight of materials and assessing human health damage using ‘disability-adjusted life years’ (DALYs) [16].

A green building material (GBM) is an ecological, healthy, recycled, or high-performance material that is cable of minimizing its impacts on the environment and human health throughout its life cycle (LC) (including resource use, manufacturing, use, operation, disposal, and recycling) [17,18]. It is specially made from non-toxic, natural, and organic substances and can reduce IAQ contaminants [19]. In fact, indoor air measurement is one of the main paths used in green building schemes to manage IAQ. The IAQ refers to indoor air quality, which has been shown by pollutants and thermal conditions to affect the health, comfort, and efficiency of occupants [20]. GBMs can help divert IAQ liability claims and meet consumer needs and regulatory requirements [19].

Further, using polymeric products such as CBMs derived from the non-renewable resources is an important cause of VOC emissions indoors [21]. The U.S. Green Building Council has recognized the chlorine content of polyvinyl chloride (PVC) building materials, and dioxin emissions consistently place PVC among the worst materials for human health. To resolve this issue, replacing CBMs with biocomposites results in reduced environmental impacts and human health concerns. Previous studies have found that biocomposites as renewable resources replace non-renewable material such as petroleum-based composite from LC insight [22,23], mostly due to indoor air contaminants, especially VOC emission [24]. Biocomposite as GBM is made of biopolymer and natural fibers [25,26] that can reduce indoor air pollutants and total impacts on the environment and human health [19,20]. However, no work has yet been done to address the role of full biocomposites as GBMs in reducing environmental and human health impacts [27,28]. Therefore, the study aimed to develop a model of a fully hybrid bio-based biocomposite as GBMs, and compare it with a common fully petroleum-based composite as CBMs. Therefore, the objectives of this research are as follows:

❖ to develop and specify biocomposite as GBMs and common petroleum-based composite as CBMs,
❖ to evaluate and contrast their human health impacts through Simapro software,
❖ to measure and compare their emissions of VOCs through small chamber test.

To achieve these objectives, the LCA method was modeled using Simapro software, and a small chamber test was used to measure the amount of VOCs emissions. The next section deals with the literature review that determines it.

## 2. Literature Review

### 2.1. Biocomposite as GBMs to Decline VOC Emissions

Sustainable development consists of several goals that coalesce into 3Ps, namely, environmental, economic, and social pillars. This unique development toward sustainability was introduced by Barbier (1987), which underlines the prospect of trade-offs among the countless economic, environmental, and social goals, with positive or negative preference [29,30].

Green or biocomposite materials are structural materials made from renewable resources that are biodegradable [31]. They are affected by bacteria, turning them into small substances without any harm to the environment [32]. The biocomposite materials are being researched and developed to replace non- and less eco-friendly materials used in the construction industry as potential candidates for the next generation of GBMs. The potential applications for biocomposite within buildings include framing, walls and wallboard, window frames, doors, flooring, decorative paneling, cubicle walls, and ceiling panels. The components of biocomposite are natural fibers as reinforcement and biopolymers as matrixes, which in fibers are stronger and stiffer than the polymeric matrix [32,33]. Totally, behaviors of biocomposites depend on certain factors, including kinds of fibers, matrix, and distribution of fibers on matrix, etc. This study addresses the natural fiber (NF) hybrid biocomposite and briefly elucidates biocomposite components.

Biopolymers are polymers derived from living organisms, such as plants and microbes. The primary sources of biopolymers are renewable, which is in contrast to petroleum [34]. Polyhydroxybutyrate (PHB) is the most common biopolymer that is considered as a matrix for biocomposites in this research. The mechanical properties are reported to be equal or even better than traditional thermoplastics [35]. PHB is an organic and biodegradable polymer [36]. The major benefits of PHB include: biodegradablity, made from a low-cost renewable carbon source, less expensive to produce from sugar or corn starch, produced with lower energy inputs, and releases lower greenhouse gas emissions over its life cycle compared to petrochemical plastic materials, and the key to a true cradle-to-cradle carbon cycle [37].

Usage of natural fibers (NFs) in biocomposites has received attention due to their relatively low price [38]. Moreover, they are recyclable and show more strength [39]. In fact, the main reasons for the increasing popularity of NFs are related to having consistent quality and being environmentally friendly [40]. NFs possess a moderately high specific strength and stiffness that can be used as reinforcing materials in biocomposites to make a practical structural composite material. Kenaf is a bast fiber that has a great potential as a reinforcing fiber in composites due to its superior toughness and high aspect ratio in comparison to other fibers. It has the highest carbon dioxide absorption of any plant (one ton of kenaf absorbs 1.5 tons of atmospheric CO_2_), a valuable tool in the prevention of global warming and priority for choosing as green materials [41]. Furthermore, the study of lignocellulose fibers has revealed that the properties of fibers can be better used in hybrid composites to use as an alternative to synthetic fiber composites [42,43]. Among all NFs, oil palm fibers (OPFs) are hard and tough and found to be a potential reinforcement in composite applications [44]. This study considered a kenaf/OPF hybrid reinforced PHB biocomposite as a green building material (GBM).

Most research and development on biocomposites had been targeting the packaging, automobile, medical, and interior-design industries [45,46]. However, some important research considered the use of biocomposites in construction applications [40,47]. Table 1 shows the most important research about biocomposites in construction, investigating the characters and roles of the bast fibers in composites and biocomposites, which revealed that these fully bio-based materials have capabilities to be appropriate to use in the construction industry (Table 1).

The GBM is an ecological, healthy, recycled, or high-performance building material that is capable of efficiently minimizing impacts to Earth’s environment and damage to human health during its entire life cycle.

So, the GBMs directly affect overall quality of life due to the decline in environmental and human health impacts. The IAQ as a GBM criteria is a term that refers to the air quality within and around buildings and structures, especially as it relates to the health and comfort of building occupants. Deterioration of IAQ results from various pollution sources and is highly related to residents’ activities and ventilation performance. It can be affected by gases (including carbon monoxide, radon, and volatile organic compounds), particulates, microbial contaminants (mold, bacteria) or any mass or energy stressor that can induce adverse health conditions (California Indoor Air Quality Program). Among the emissions from interior finish materials, formaldehyde (HCHO) and volatile organic compounds (VOCs) are the main substances subject to evaluation by all IAQ certification organizations [58].

There are various inconsistent criteria and variables in terms of material selection, including the local availability of natural materials, high performance with various terms such as thermal and strength, cost, low energy consumption, eco-friendly, and aesthetic. People spend over 80% of their time indoors [59], and pollutants from interior finishing or non-structural building materials have a major impact on air quality and can affect occupants’ health [60,61,62]. The finishing building materials can produce health problems with some indoor air deterioration such as that caused by VOCs [60,63]. Therefore, careful selection of materials can improve air quality, healthy, and comfortable indoor environments [64]. In this regard, previous research has been done for different parts of non-structural building materials including floor [65,66], wallpaper [67,68], insulation [69,70], adhesive [71], paint [72], and wood-based panels [73]. However, the current research focused on a specific biocomposite as GBM especially fabricated from non-toxic, natural, and organic materials that could reduce IAQ contaminants and the accompanying complaints and claims. GBMs can help divert IAQ liability claims, respond to consumer demand, and provide for compliance with certain regulatory requirements [63]. However, there is no perfect GBM due to a lack at both the principle and product development levels [4].

### 2.2. Life Cycle Assessment (LCA): Software and Human Health

The impacts of CBMs on the environment and human health have been compassed from the use of raw materials during construction, maintenance, and renovation to the emission of harmful substances through their life cycle [74]. The LCA method provides guidelines for materials selection that quantifies and compares inflows of the inputs, outputs, and the potential environmental impacts of the product system throughout its life cycle [75]. This approach is the only appropriate method for comparison of CBMs with an alternative that can lead to a reduction in the overall environmental burdening and human health impact from the construction industry [76,77], which has been used in construction division since 1990. Although LCA is a complex and expensive methodology, the progression of LCA software leads to resolve the complexity of this method in material science.

**Figure 1:**
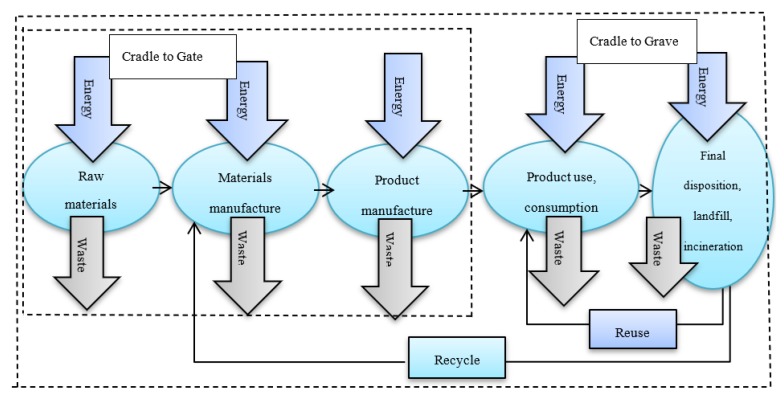
Life cycle (LC) of products from cradle to grave.

The Society of Environmental Toxicology and Chemistry established the LCA methodology formerly in the aim of reducing resource consumption and environmental burdening of products [78,79,80]. Principally, there are four major steps of an LCA, which include: goal and scope, inventory analysis (LCI), impact assessment (LCIA), and interpretation [81]. The LCA method provides guidelines for materials selection, which quantifies and compares inflows of materials and energy and outflows of emissions of materials on an LC perspective for possibilities of improvement. Figure 1 shows the LC of products from the cradle to the grave.

The LC includes all the stages of a product’s life from the cradle to the grave (i.e., from raw material extraction through materials processing, manufacture, distribution, use, repair and maintenance, and disposal or recycling). Furthermore, the partial product LC from resource extraction (cradle) to the factory gate is named LC from cradle to gate. LCA is an actual implementation to evaluate the environmental impact of the materials and products. There are different methods for life cycle impact assessment (LCIA) such as CML 2000, ReCiPe, and EPS 2000. These methods have been settled to determine the impact of releases of damaging constituents on human health, which are considered to make allowance for outdoor sources of pollution, not indoor ones.

**Figure 2:**
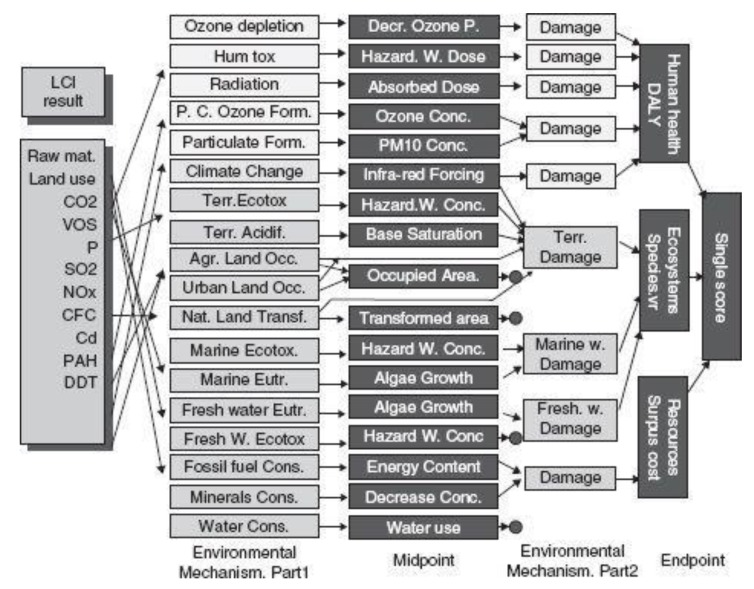
The overall structure of ReCiPe methodology [85]. LCI: life cycle inventory; DALY: disability-adjusted life year.

Recently, LCA is considered more than previously, and this led to the development of methods, software, and databases for the execution of LCA [82]. There are about or more than 40 LCA programs that can be divided according to their use as educational or commercial. Common brand software tools include OpenLCA and GaBi from Germany, SimaPro from the Netherlands, TEAM from France, etc., and they provide a framework for improving and ensuring the choice of materials [83]. SimaPro is the most widely used LCA software that offers standardization as well as the ultimate flexibility, providing an overview of the potential impact any design will have under realistic conditions [84]. This study considered it as the main software for analyzing LCA because it contains a number of impact assessment methods to calculate impacts on environmental and human health. The ReCiPe method is considered in this research due to combining the benefits of two methods, including “the problem-oriented approach at a midpoint level” from the CML-IA method and “the damage-oriented approach at an endpoint level” from Eco-indicator 99 method (Figure 2). Figure 2 determines the relationship between life cycle inventory (LCI) parameters (left), midpoint indicator (middle), and endpoint indicator (right) in the ReCiPe method [85]. The ambiguity of the complex results from the CML-IA method with 18 categories at the midpoint is relatively low. However, the damage oriented approach of Eco-indicator 99 at the endpoint makes the interpretation of the results easier with only three impact categories.

The ReCiPe encompasses these two groups of impact categories. In the endpoint level, most of the midpoint impact categories are reproduced by damage factors and aggregated into three endpoint categories: human health, ecosystem, and resource cost. The three endpoint categories are normalized, weighted, and aggregated into a single score.

The concept for damage assessment to human health in the LCA procedure is based on “disability-adjusted life years” (DALYs), which was introduced by Hofstetter in 1998 [86]. The values for DALYs have been stated for a wide range of diseases, including various cancers, vector-borne diseases, and non-communicable diseases [87,88,89]. In ReCiPe, the DALYs concept includes years of life lost and years of life disabled, without age weighting and discounting, as a default setting for quantifying the damage contributing to the human health area of protection within LCA.

### 2.3. VOC Emissions

VOCs are a large group of carbon-based chemicals that easily evaporate at room temperature. While most people can smell high levels of some VOCs, other VOCs have no odor. Odor does not indicate the level of risk from inhalation of this group of chemicals. There are thousands of different VOCs produced and used in our daily lives [90]. Breathing low levels of VOCs for long periods may increase some people’s risk of health problems [91,92]. Common symptoms of exposure to VOCs include:

❖ Short-term (Acute) exposure to high levels of VOCs: eye, nose and throat irritation, headaches,- nausea/vomiting, dizziness, worsening of asthma symptoms,
❖ Long-term (Chronic) exposure to high levels of VOCs: increased risk of cancer, liver damage, kidney damage, and central nervous system damage.

There are various agency standards regarding the demonstration of common indoor pollutants and their standards listed in Table 2. Table 3 shows the illustration of major indoor air pollutants and their negative affects [81,93,94].

Emission-based certification systems for interior finish materials are divided into grade certification or suitability determination depending on whether emission amounts exceed certain criteria. Some countries’ standard program sets LCA standards for environmental impact across life cycle encompassing pollutant emissions and production, distribution, recycling, and disposal of interior finish materials such as Green Guard in the USA, Eco-Labeling program in South Korea, etc.

The VOCs are a significant class of indoor air pollutants, with indoor attentions generally higher than outdoors. Furthermore, formaldehyde is a priority VOC because of its frequent occurrence in indoor air and the serious health outcomes resulting from exposure [19]. Recently, the attentiveness of health risks linked with hazardous indoor air pollutants has activated a growing public health concern. Moreover, the emissions feature of building materials has been widely reported [95]. Böhm et al. (2012) studied formaldehyde emission (FE) monitoring from a variety of solid wood, plywood, block-board, and flooring products manufactured, which are used for building and furnishing materials [96]. They reported the differences in the FE values for various wood products. Based on the results, in the first week after manufacturing, the FE was high; however, the decrease in FE was noticeable at the two-week measurement for all of the materials, especially for the painted block-boards [96].

Numerous large-scale studies have also been directed in existing homes to quantify contaminant attentions. In some cases, information was simultaneously collected about potential contaminant sources. Based on researches, wet building products such as paints and adhesives contributed more significantly to VOC levels measured indoors [97,98]. Chuck and Derrick (1998) reviewed the VOC emission from polymeric materials used in buildings [99]. The study highlighted that polymeric materials such as vinyl floorings, carpets and underlays, adhesives, wall-covering materials, caulks, sealants, thermal insulating materials, paints, coatings and varnishes, and waterproofing membranes and bituminous emulsions are important sources of VOC emissions in buildings [99].

Kim et al. (2006) evaluated the VOC emissions from building finishing materials using a small chamber and VOC analyzer [58]. The research indicated that emissions of VOCs from wood-based composites could adversely affect indoor air quality. They endorsed the desiccator and chamber method for VOC analysis as a good alternative to the traditional chamber method for determining VOC emission levels from building products. Lee et al. (2012) focused on finishing material management systems for indoor air quality of apartment buildings and aimed to carry out research on a system for the selection of apartment house finish materials based on IAQ performance evaluation [100]. The result revealed that it is very important to control and evaluate the pollutant generation through the selected finish materials in buildings for preventing IAQ deterioration. Ayrilmis et al. (2016) tried to investigate the formaldehyde emission and total volatile organic compounds (TVOCs) emitted from the laminated veneer lumber (LVL) produced as building materials with the different-grade Urea formaldehyde (UF) resins that were modified with different amounts of the micro-fibrillated cellulose (MFC) using a thermal extractor [101]. They encountered the highest VOC emitted from the LVLs that were found to be toluene, followed by xylene, benzene, and ethyl-benzene, respectively. The TVOC from the LVLs considerably decreased with increasing MFC content, and usage of MFC in the UF resin was highlighted as an environmentally friendly solution for reducing the TVOC from the wood-based panels.

In terms of green or biocomposite materials, Lee et al. (2008) investigated biocomposites’ formaldehyde and TVOC emission [24]. Based on the result, the TVOC emission level is very low in all of the biocomposite samples except the formaldehyde and TVOC emission level of the bio-composites with the attached veneer. Cheng et al. (2015) compared conventional and green building materials in term of VOC and carbonyl emissions [63]. The research result showed that GBMs had lower emissions than conventional building materials, especially for wooden flooring and gypsum board.

Therefore, building materials need to be evaluated with respect to their human health impacts and VOC emissions. Based on the above literature review of previous studies in terms of outdoor and indoor impacts form non-structural building materials, this study hypothesized that biocomposite samples as GBMs have the potential for reducing human health impacts and VOC emission.

## 3. Materials and Methods

### 3.1. Materials: Constituents and Preparation

This study considered hybrid kenaf with oil palm fibers (OPFs) in terms of reinforcement for target and goal biocomposite. The glass, kenaf, and oil palm fiber mats were obtained from Innovative Pultrusion Sdn Bhd Company, Malaysia. Moreover, the polyethylene (PE), polypropylene (PP), and polyhydroxybutyrate (PHB) granules were obtained from Goodfellow Cambridge Ltd. company, England, UK. Table 4 shows the various properties among PHB and two other polymers, PP and PE, based on the Goodfellow website information [102]. Table 4 compares significant characters and shows some similarity in physical and mechanical properties of PHB, PP, and PE.

**Figure 3:**
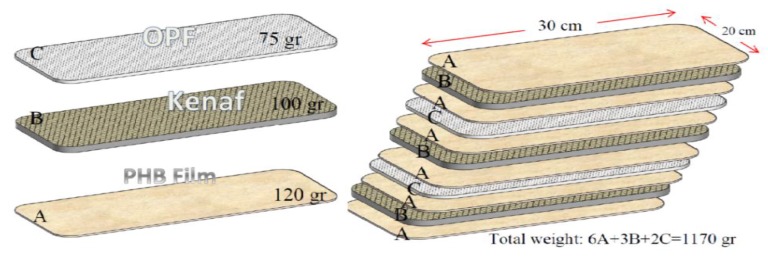
Sample arrangement of kenaf/OPF (oil palm fiber) hybrid PHB biocomposite.

Composite manufacturing methods vary based on composite form, the fiber type, and the matrix type. Additionally, manufacturing method and volume fraction greatly affect biocomposite behavior. Heat and pressure are usually applied for manufacturing composites. In this study, based on the compression molding and laminate method, the biocomposites were made from polymer films and kenaf and OPF fabric. Preparation of the kenaf–OPF hybrid PHB biocomposite plate required two steps, PHB film and NF preparation. Previous research has developed various specific biocomposites in terms of mechanical properties, which determined the best layer arrangement for hybrid biocomposites [103]. The tensile and flexural test of woven kenaf bast fibre/oil palm empty fruit bunches (KBFw/EFB) hybrid reinforced PHB biocomposite with 11 layers revealed that this sample has the capability to replace with some wood and woody production as non-structural building materials. Figure 3 shows the sample layout of hybrid biocomposite with 11 layer laminate (three layers kenaf mat, two layers OPF mat, and six layers PHB film). The sample arrangement has variety in reinforcement and matrix percent. In this study, based on mechanical properties of biocomposite; the percentage of NFs/biopolymer is around (33%/67%), and the percentage of kenaf/OPF is around (33%/67%). For the preparation of GFPP and GFPE, the percentages of fiber and polymer are the same.

### 3.2. Methods

The methodology framework of this research is divided into two parts: first SimaPro software modeling with the ReCiPe method for evaluation of human health impacts based on LCA methodology, and secondly, small chamber test (ASTM-D5116) for VOC investigation.

#### 3.2.1. Outdoor Impact Assessment to Human Health with SimaPro Software

LCA is a complex and expensive methodology. The evolution of LCA software leads to resolving the complexity of this method in material science. SimaPro is an LCA software that was used to quantify the total amount of human health impact in this part of the study. It is very important to recognize and simplify the complex reality of materials production during life cycle analysis. SimaPro software has the capability to quantify and compare inflows of embodied materials and outflows of emissions from materials on a life cycle perspective. The most important assumptions and limitations for this part of the methodology include:

❖ The functional unit is an important issue in product comparisons, which should be defined for ensuring a common basis in terms of comparing two products. In this study, the functional and declared unit is set to be one kilogram of output.
❖ The initial system boundary is a helpful way to draw and determine a diagram and boundary for products. Therefore, the system boundary applied in software modeling focuses on ranges from cradle to gate.

Definitely, biocomposite is a biodegradable material that needs less energy for recycling during the end-of-life, compared with petroleum-based composite. In this case, the impacts regarding installation and maintenance are negligible and the use phase and end-of-life are not included in the system boundary. Therefore, the system boundary for this research is focused on cradle to gate. Cradle-to-gate is a valuation of a restricted material life cycle from resource extraction (cradle) to the factory gate, before it is transported to the customer. The use phase and disposal phase of the product are omitted in this case.

In addition, the life cycle inventory (LCI) for the acquisition of raw materials information is achieved from Eco-invent, Industry data, and U.S. Life Cycle Inventory Database (USLCI) libraries [104,105,106]. Totally, SimaPro contains a number of impact assessment methods, which are used to calculate impact assessment results. In this research, the ReCiPe method was considered for LCIA form SimaPro methods’ library, that it was created by Netherlands National Institute for Public Health and the Environment (RIVM), institute of the Faculty of Science of Leiden University (CML), PRé Consultants, Radboud Universiteit Nijmegen, and CE Delft [85]. ReCiPe is the most recent and harmonized indicator approach available in LCIA. In ReCiPe, the user can choose midpoint indicators or endpoint indicators for interpreting for this quantitative list of emissions [107]. Under the endpoint approach, total impacts are grouped into general issues of concern such as human health, natural environment, and resources [108,109].

#### 3.2.2. Indoor Impact Assessment to Human Health with Small Chamber Test

**Figure 4:**
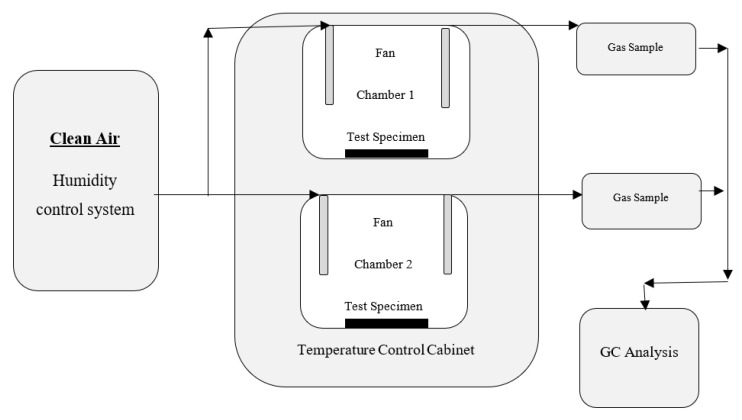
Small chamber test facility schematic.

This part of the study focused on concluding the emissions of organic compounds from indoor materials with small-scale environmental test chambers. The evaluation of indoor air pollution from bio-based and petroleum-based composite as non-structural building materials is measured in conformity with the ASTM designation: D5116-10 [110]. Experimentally, a small chamber technique is available for evaluating organic emissions from indoor materials in the building. A facility considered and functioned to control organic emission rates from building materials includes test chambers, clean-air generation system, monitoring and control systems, sample collection and analysis equipment, and standards generation and calibration systems. Figure 4 is a schematic showing an example system with two test chambers. The clean air–humidity control system has an air providing part, a humidifier, and a ventilation system to cleanse the air.

The sample size of building materials can range from a few liters to a few cubic meters. Moreover, the basic experimental design for small chamber tests should include and test the effects of various parameters on the emission characteristics of the materials. Six variables are generally considered to be critical parameters: temperature [T], humidity [H], air exchange rate [N], product loading [L], time [t], and air velocity [v]. Before starting the chamber test, it should be washed with distilled water and heated at 260 °C to eliminate any pollutants from the chamber. Table 5 shows the test conditions in the 20 L small chamber method. Additionally, two stainless flame seal packets are used during the test, in which every sealed box just allows emission from one surface of every sample, and not from the edges [111].

**Figure 5:**
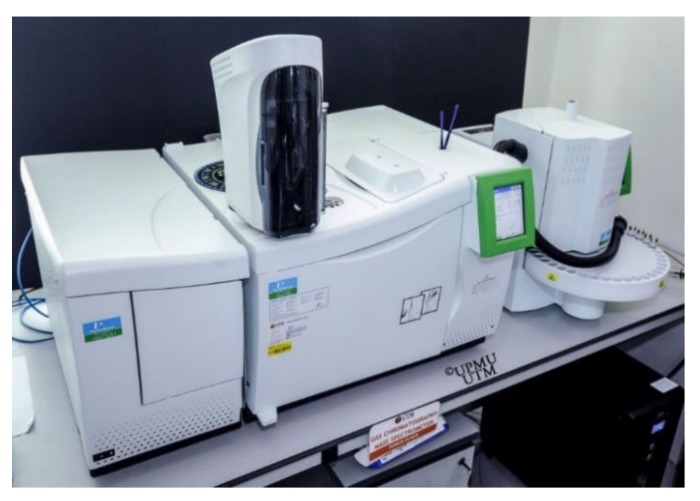
Gas chromatography–mass spectrum (GC-MS) instrument.

The samples are collected after 1, 3, 5, and 7 days using Tenax-TA tubes from the air outlet the bake-out chamber. The total volatile organic compounds (TVOCs) concentrations were analyzed by gas chromatography with a mass spectrum (GC-MS) (Figure 5). It is an analytical method that combines the features of gas chromatography and mass spectrometry to identify different substances within a test sample [112]. The TVOC value is not only the amount of the volatile organic compounds distinguished in an analysis. This value of a sample is determined by the integration of the chromatographic peak area between C6–C16 with a 100 ng total integrated area toluene peak comparison calculated.

## 4. Results and Discussions

### 4.1. Results and Discussion for Outdoor Human Health Impacts

The objective of this part of the research focused on terms of human health from comparing LCIA of bio-based with petroleum-based composites. The SimaPro outcome for LCIA encompasses two levels of impact categories: midpoint and endpoint level. The greatest of midpoint impact groups and indicators are reproduced midpoint indicators by damage factors. Damage assessment converts midpoint indicators into three endpoint categories: ecosystem, human health, and resources with different units. Furthermore, there are three different indicators (damage assessment, normalization, and weighting) in endpoint level outcomes of impacts assessment through SimaPro software with the ReCiPe method.

Damage assessment was appended to make use of “endpoint methods”, such as Eco-indicator 99 and the EPS2000. Damage assessment aims to mix a number of impact category indicators into a damage category. The damage category is called the area of protection (AoP) and includes ecosystems, resources, and human health. In ReCiPe, the AoP of human health has been represented by the endpoint category “damage to human health”, which combines mortality and morbidity. The AoP of the natural environment was represented by the loss of species, and the increased set of future extractions represented the AoP of natural resources.

**Figure 6:**
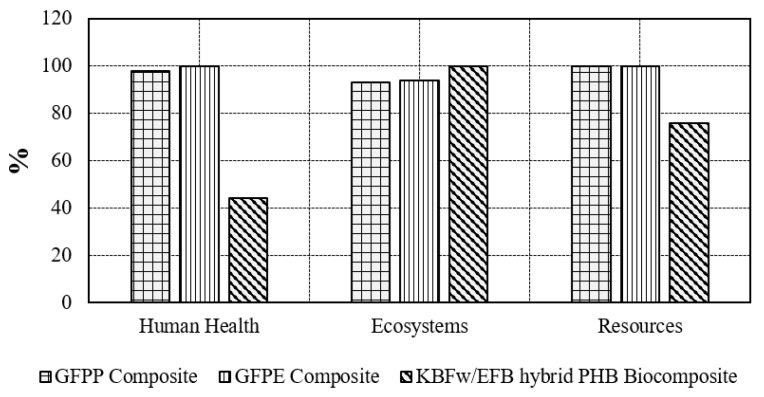
Damage assessment indicator.

Figure 6 shows the damage assessment indicator result from the endpoint characterization factors used in the ReCiPe method. They are displayed and plotted on a 100% scale. The damage category is called an area of protection, which is including ecosystem, resources, and human health, with a different unit. The unit of damage to human health is named DALYs, which means (disability-adjusted life years). In fact, damage to human health expressed as the quantity of years of life lost and the quantity of years lived disabled.

**Figure 7:**
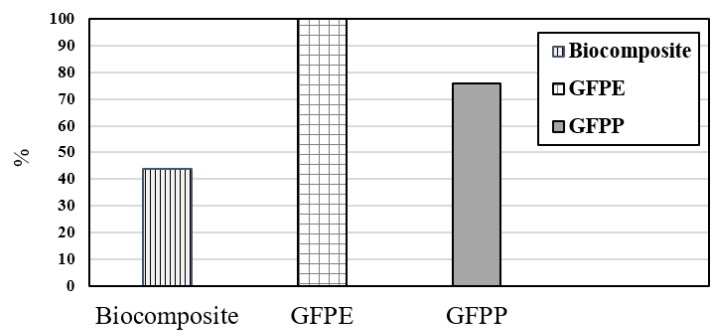
Damage assessment indicator to human health.

Therefore, these endpoint outcomes can assist in extending discussion in terms of human health impact. The concept of DALYs has proven to be a useful metric in the assessment of human health damage in LCA [113]. Based on Figure 7, the amount of damage to human health declined on the substitution of bio-based composite for petroleum-based composite (GFPP, and GFPE) from 100% to 44%.

Further impact assessment indicators from damage oriented scores are the normalization and weighting that simplify the complex interpretation of the results in midpoint level and permanently considered them on the basis of the endpoint characterization. Normalization is a method to demonstrate at what level an impact category contributes to the overall environmental and human health problems. Normalization also solves the incompatibility of units. It shows to what extent an impact category indicator consequence has a quite high or low value compared to a reference. The normalization provides comprehensible results for comparing the impact of two products with the same unit. During the procedure of normalization, when emissions per year are used, the exact unit of a normalized value is a year.

**Figure 8:**
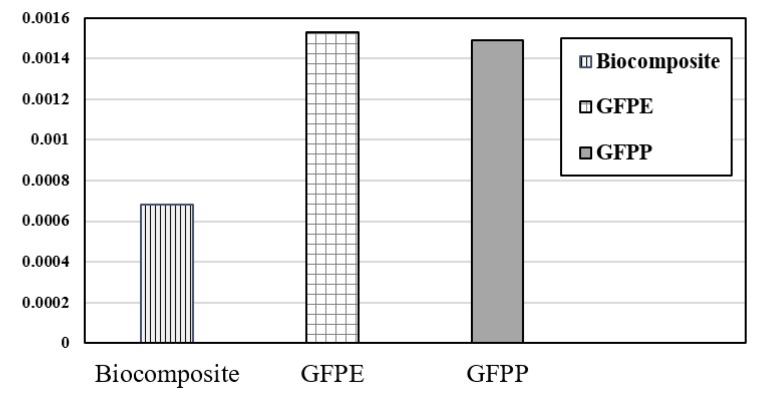
Normalization indicator.

Based on normalization results (Figure 8), the impact category on human health declined noticeably from 0.0015 to 0.00065 on substitution of petroleum-based composite (GFPP and GFPE) with fully biocomposite materials.

Weighting is the greatest provocative and tough step in life cycle impact assessment, particularly for midpoint approaches, which is used quite comprehensively for internal decision-making. The value of weighting is mPt. One point is equivalent to 1/1000 of average Europeans’ environmental impact in one year, and 1 mPt (mili point) is equal 1/1000 Pt. Totally, weighting presents LCA results as a single score, which allows you to easily compare the human health impact of two different products.

**Figure 9:**
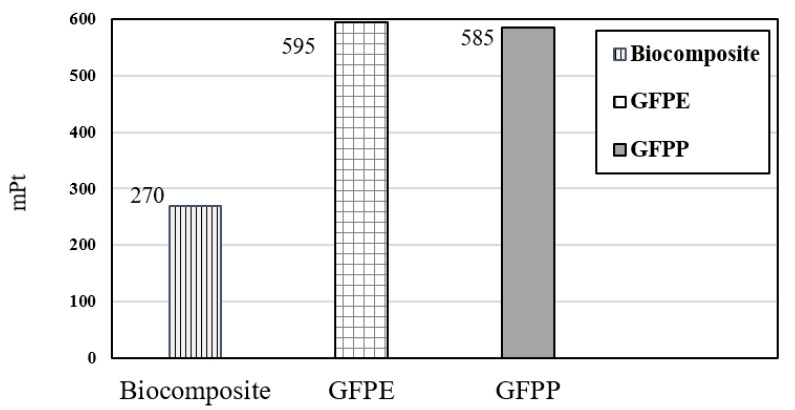
Weighting indicator.

Figure 9 shows the weighting indicator, which determines the significant effect of this research strategy for human health gauge. The impacts on human health reduce from 600 to 270 mPt due to the substitution of petroleum-based composite with biocomposite. Therefore, the SimaPro analysis shows that the total impact on human health declined around half with the substitution of biocomposite to petroleum-based composite (GFPP and GFPE).

### 4.2. Result and Discussion from the Small Chamber Test for Indoor Human Health Impacts

The 20 L chamber test method is commonly used for the assessment of TVOCs and formaldehyde emissions based on ASTM standards. The results of this process emphasized that small chamber evaluations are used to determine source emission rates for comparison between petroleum-based composite with biocomposites. These rates are then used in IAQ models to predict the indoor concentration of the compounds emitted from the tested samples.

The gas chromatography/mass spectrometry (GC/MS) instrument separates chemical mixtures (the GC component) and identifies the components at a molecular level (the MS component). It is one of the most accurate tools for analyzing environmental samples. The GC works on the principle that a mixture will separate into individual substances when heated. The heated gases are carried through a column with an inert gas. As the separated substances emerge from the column opening, they flow into the MS. Mass spectrometry identifies compounds by the mass of the analyzed molecule.

Automated Mass Deconvolution and Identification System (AMDIS) software is supplied by the National Institute of Science and Technology (NIST) with the library package. The software de-convolutes the spectra of overlapping chromatographic peaks and pulls out “clean target spectra” from overlapping peaks. The TVOC value of a sample is determined by the integration of the chromatographic peak area between C6–C16.

**Figure 10:**
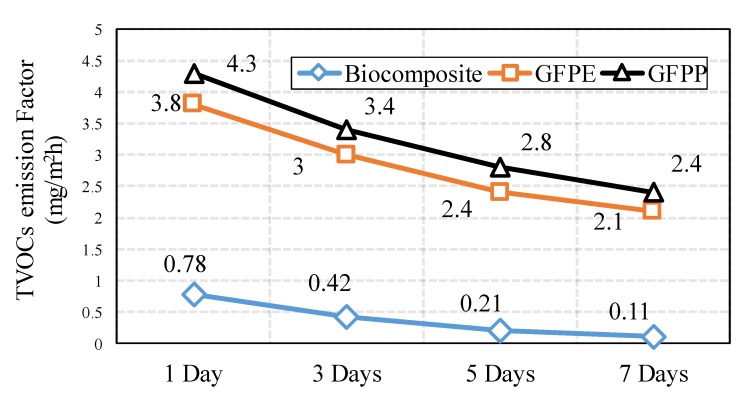
Total volatile organic compound (TVOC) emission factor (mg/m^2^h) of different samples during seven days of monitoring.

Figure 10 shows the TVOCs emissions, which were detected 1, 3, 5, and 7 days after the preparation of the sample. According to these results, the TVOCs emissions of all of the samples declined during the seven days of monitoring. Biocomposite has lower emission of TVOCs in compare to other materials. Moreover, the difference in TVOC emission between biocomposite and petroleum-based composite was very high. The amount of TVOCs of biocomposite monitor from 0.78 (mg/m^2^h) on the first day and gradually decreased until 0.11 (mg/m^2^h) in seven days. However, the TVOCs emission of GFPP was higher than that of GFPE; the amount of VOCs from both sharply decreased during the seven days of monitoring. The higher rate TVOCs emissions belonged to GFPP on the first day (around 4.3 (mg/m^2^h)), and its sharply declined around 50% for seven days to 2.4 (mg/m^2^h).

According to the literature review about various standards related to TVOCs, there is a difference between the generic standard of chamber properties during the test and rating for evaluation the rate of TVOC emissions for building products. For example, the European test methods are based on ISO 16000 [110] such as AgBB (Germany) [114] and AFSSET (France) [115], and in the U.S., Californian CHPS specification [116], also known as Section 01350, which is based on ASTM-D5116 [117]. Therefore, there is a different rate of consideration as limited values for comparison of emission rates. The total VOC emission limitation and the acceptable one for the building is different: AFSSET and AgBB: 1250 (μg/m^2^h) and CHPS: around 1000 (μg/m^2^h). Each milligram/liter (mg/m^2^h) equals 1000 microgram/liter (μg/m^2^h).

Based on Figure 10, the TVOC emission rate in seven days equals 2400 μg/m^2^h for GFPP, 2100 μg/m^2^h for GFPE, and 110 μg/m^2^h for biocomposite. The TVOC emissions of both petroleum composites (GFPP and GFPE) was higher than the acceptable rate of emissions based on AFSSET, AgBB, and CHPS standard. Therefore, the egregious difference of TVOCs emissions between biocomposite and petroleum composites, with acceptable standards rate, could highlight the biocomposite’s function in terms of indoor air quality.

## 5. Conclusions

The impact of building materials on human health is unavoidable. However, the movement of conventional building materials to green materials tried to reduce the total impacts on human health in indoor and outdoor. Green building materials (GBMs) with non-toxic, natural, and organic compounds have the potential to reduce indoor air quality (IAQ) deterioration and total impacts on human health. Green composite or biocomposite as GBMs are bio-based, healthy, and recyclable, which progress the total quality of life. By using small chamber test (ASTM-D5116), for VOCs investigation, and SimaPro software modeling with ReCiPe method, for evaluating human health impacts based on Life Cycle Assessment (LCA) methodology, this study tried to develop a model of fully hybrid bio-based biocomposite as non-structural GBMs and compared it with fully petroleum-based composite in term of volatile organic compound (VOC) emissions and human health impacts. The results recommend substituting the fully petroleum-based composite with the fully hybrid bio-based biocomposite, which can significantly decline the rate of impacts on human health in terms of indoor and outdoor.

Based on the results, the green or biocomposite as GBMs with non-toxic, natural, and organic compounds considerably demoted indoor air quality (IAQ) deterioration and total impacts on human health, while this was not observed from petroleum-based composite. In terms of outdoor impacts on human health, the result transfigured the life cycle inventory of these composites with SimaPro software to create a certain level of damage with a single score. The results revealed that the total outdoor impacts on human health decrease around one third with substitution of biocomposites for petroleum-based composites based on LCIA. In terms of indoor impacts on human health, the result developed based on TVOC emissions from petroleum-based composites and biocomposite, with a 20 L chamber test method (ASTM-D5116). The obtained result exposed that the total indoor impacts of TVOCs on human health incredibly decline with the substitution of biocomposites for petroleum-based composites. The TVOC emission rate from biocomposites is acceptable according to a different standard (such as AgBB, AFSSET, and CHPS), but this is not true for petroleum-based composites.

This study provides significant coordination between the development of biocomposite principles as GBMs and the level of product development in terms of VOCs. Additionally, this study affords an essential orientation and the first phase for future investigation to discuss the role of different biocomposites in reducing environmental and human health impacts.

## Figures and Tables

**Table 1:** Bast fiber-reinforced biocomposites that are recommended as building materials.

No.	Biocomposite	Results	References
1	Hemp fiber/cellulose acetate composites	Examined the base for rheological, thermal, and morphological characteristics	[48]
2	Hemp fiber/cellulose acetate composites	Examined base for physico-mechanical and thermo-mechanical properties	[49]
3	Hemp-reinforced biocomposites	Comparable properties with wood and woody products in construction industry	[48,49,50]
4	Hemp cellulose acetate/PHB biocomposites	Have mechanical properties similar to structural wood	[50]
5	PHB and various co-polymers of PHB combined with hemp fiber	Show promise for use in construction due to their good mechanical characteristics. They emphasized that biocomposites have the potential to be used for scaffolding, formwork, flooring, walls, and for many other applications within buildings, as well as temporary construction.	[51,52]
6	PHB and various co-polymers of PHB combined with flax fiber		[53,54]
7	PHB and various co-polymers of PHB combined with jute fiber		[55,56]
8	PHB and various co-polymers of PHB combined with kenaf fiber		[57]

**Table 2:** International organizations involved in air quality standards.

No.	Country	Organization	Web Address
1	Worldwide	World Health Organization	http://www.who.int/en/
2	U.S.	U.S. Environmental Protection Agency	http://www.epa.gov/
3	Canada	Health Canada	www.hc-sc.gc.ca
4	Europe	European Commission	http://ec.europa.eu/index_en.htm
5	UK	Health and Safety Commission	http://www.hse.gov.uk/
6	Australia	National Health and Medical Research Council	https://www.nhmrc.gov.au/
7	Singapore	Singapore Indoor Air Quality Guideline	http://www.nea.gov.sg
8	Malaysia	Department of Occupational Safety and Health	http://www.dosh.gov.my/index.php?lang=en
9	Korea	Korea Environmental Industry and Technology Institute	http://www.keiti.re.kr/en/index.do
10	China	State Environment Protection Agency	http://www.sepa.gov.cn/

**Table 3:** Major indoor air pollutants and their negative effects. VOCs: volatile organic compounds.

Pollutants		Negative Effects of Pollutants
VOCs	Benzene	Bone marrow damage, thrombopenia, leukopenia, anemia
	Toluene	Poisonous to the liver, blood, nerve, fatigue, mental storm: strongest toxicity
	Xylene	Extremely toxic to the nervous system
	Ethylbenzene	High levels of toxicity for the nervous system
	Styrene	Acute toxicity, irritating the mucous membrane of the eyeball, shriveling the central nervous system
HCHO		Irritation to the eyes, nose, throat, cough, diarrhea, vertigo, nausea, skin disease, rhinitis, emotional instability, losing memory, damaging the nervous system, carcinogenesis

**Table 4:** The Goodfellow Cambridge Ltd. company’s information about properties of PHB, PP, and PE.

Properties	PHB	PP	PE
Chemical Resistance			
Acids—dilute Alcohols Alkalis Greases and oils	Fair Fair Poor Good	Good–Fair Good Good Good–Fair	Good Good Good Good–Fair
Mechanical Properties			
Elongation at break (%) Izod impact strength (J m^−1^) Tensile modulus (GPa) Tensile strength (MPa)	6 35–60 3.5 40	150–300 20–100 0.9–1.5 25–40	500 >1000 0.2–1.2 20–40
Physical Properties			
Density (g cm^−3^) Resistance to ultraviolet	1.25 Fair	0.9 Poor	0.94 Poor
Thermal Properties			
Upper working temperature (°C)	95	90–120	55–95
Biodegradability	Yes	No	No

**Table 5:** Test conditions in the 20 L small chamber method.

Variables	Condition
Chamber volume	20 L
Sample size	(0.15 m × 0.15 m × 2) = 0.045 m^2^
Air flow rate	0.01 m^3^/h
Ventilation rate	0.5 /h
Sample loading factor	(0.045 m^2^/0.02 m^3^) = 2.25 m^2^/m^3^
Temperature	23 ± 1 °C
Relative humidity	50% ± 5%
